# Supplementary material for: Identifying opportunities to optimize mass drug administration for soil-transmitted helminths: A visualization and descriptive analysis using process mapping
Source: PLoS Negl Trop Dis. 2024 Jan 4;18(1):e0011772. doi: 10.1371/journal.pntd.0011772 (PMC10793904; doi:10.1371/journal.pntd.0011772)
Supplement: S1 Text — Worksheet used by clusters to identify activities for process maps and set ideal goal and timelines. (DOCX) [file pntd.0011772.s001.docx]

**In-depth Process Mapping Activity Worksheet**

**Country**: ________________________ **Cluster ID:** _________________ **Date:** _____________

**Cluster Process Mapping Lead Name:** _____________________­­­___ **Mobile Phone:** __________________

**Cluster Lead Name** (if different)**: ____________________________ Mobile Phone:_________________**

| **Working Group Participants** | |  |  |
| --- | --- | --- | --- |
| **Name** | **Job Title** | **Affiliation**  *(DeWorm3, MOH, MOE)* | **Background***  **(***CBD/SBD/LF***)** |
|  |  |  |  |
|  |  |  |  |
|  |  |  |  |
|  |  |  |  |
|  |  |  |  |
|  |  |  |  |
|  |  |  |  |
|  |  |  |  |
|  |  |  |  |
|  |  |  |  |
|  |  |  |  |
|  |  |  |  |
|  |  |  |  |
|  |  |  |  |
|  |  |  |  |
|  |  |  |  |
|  |  |  |  |
|  |  |  |  |
|  |  |  |  |
|  |  |  |  |

* For MOH and MOE personnel: If you are involved in school-based deworming, write SBD. If you are involved in community-based deworming, write CBD. If you were involved in previous LF campaigns, write LF. You can write more than one role if you are involved in more than one activity.

**INTRODUCTION**

Process mapping is used to visually demonstrate all of the key activities that take place to deliver a specific intervention. A process map outlines the flow of activities, materials, or information required to achieve an optimal output, such as high MDA treatment coverage. This document outlines all of the necessary steps for completing the in-depth process mapping worksheet.

Before beginning the exercise, ensure that the participating process mapping working group in this cluster is comprised of:

- Members from the DeWorm3 local site implementation science team
- Cluster lead/supervisor from each cluster
- Designated process mapping lead for each cluster (if different than cluster leader)
- Select personnel from the Ministry of Health (MOH) and Ministry of Education (MOE) or relevant community based organizations (CBOs) who are familiar with the MDA planning and delivery process for school-based and community-based deworming in this specific cluster
- There should be 5-10 individuals on the process mapping working group

The group should read through this entire worksheet before starting in order to understand the process mapping activity, including activities that will occur at follow-up.

Necessary materials include:

- In-depth process mapping worksheet
- Flip chart paper
- Markers
- Loose sheets of A4 paper
- Writing materials (pencils preferred)
- Sticky notes (6 colours – green, pink, orange, blue, purple, yellow)
- Tape

**PART 1: BASELINE ACTIVITY LIST**

**Materials**

- In-depth process mapping worksheet
- Loose sheets of A4 paper
- Writing materials (pencils preferred)
- Sticky notes (6 colours – green, pink, orange, blue, purple, yellow)

**Instructions**

**Step 1:** In Table 1, brainstorm as a group all of the activities that must take place and that you plan to perform in order to successfully deliver MDA for STH with high validated treatment coverage.

- Treatment coverage is defined as the number of people in a cluster who are treated with albendazole divided by the entire population of the cluster that is eligible for treatment. High treatment coverage is coverage ≥90%.
- In DeWorm3 intervention clusters, process mapping should be of community-wide MDA with high treatment coverage
- In DeWorm3 control clusters, process mapping should be of school-based MDA with high treatment coverage
- If more space is required, please continue on a separate sheet of paper or photocopy Table 1 as needed.
- You do not need to account for research activities (ex. conducting cross-sectional surveys), rather you should account for all activities that are required for routine MDA delivery.
- Please include even small activities (ex. drivers perform vehicle maintenance prior to MDA distribution). The goal is to understand all of the hard work and many activities that make delivering MDA with high treatment coverage possible.

**Step 2:** Record which individuals are responsible and which are involved in accomplishing each activity.

- Responsible individuals are those who are tasked with ensuring that the activity is completed.
- Involved individuals play a role in making sure the activity is completed, but are not ultimately responsible. In some circumstances there may only be responsible individuals, and no additional involved individuals.
- Please do not write specific individual’s names, but rather their job titles.

**Step 3:** Record what category most appropriately described the category.

- Activity categories include:
  - Drug supply chain
  - Training
  - MDA delivery
  - Community sensitization
  - Planning
  - Other (specify)
- Please only select one category for each activity. Choose the category that you think best encompasses the specific activity.

**Step 4:** As you write each activity in Table 1, one person from the group should also write the activity on a sticky note. Each sticky note should only have one activity written on it.

- - Use pink sticky notes if the activity is part of drug supply chain management
  - Use orange sticky notes if the activity is part of any training exercise
  - Use blue sticky notes if the activity is part of MDA delivery
  - Use green sticky notes if the activity is part of community sensitization activities
  - Use purple sticky notes if the activity is part of MDA planning
  - Use yellow sticky notes if the activity is cross-cutting, or not specific to any of the above (i.e. “other” category).


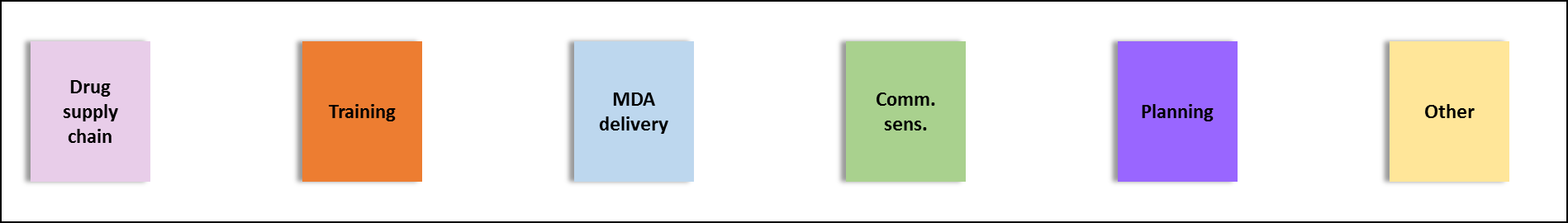


| **Table 1: Activity List** | | | |  |
| --- | --- | --- | --- | --- |
|  | **Step 1** | **Step 2** | **Step 3** | |
| **#** | **Activity** | **Who is responsible/involved** | **Category**  Drug supply chain, Training, MDA delivery, Community sensitization, Planning, Other (specify) | |
| 1 | *Example: Contact village leaders to notify about upcoming MDA campaign* | *Responsible: Cluster leader Involved: District NTD Coordinator* | *Community sensitization* | |
| 2 |  |  |  | |
| 3 |  |  |  | |
| 4 |  |  |  | |
| 5 |  |  |  | |
| 6 |  |  |  | |
| 7 |  |  |  | |
| 8 |  |  |  | |
| 9 |  |  |  | |
| 10 |  |  |  | |
| 11 |  |  |  | |
| 12 |  |  |  | |
| 13 |  |  |  | |
| 14 |  |  |  | |
| 15 |  |  |  | |
| 16 |  |  |  | |
| 17 |  |  |  | |
| 18 |  |  |  | |
| 19 |  |  |  | |
| 20 |  |  |  | |
| 21 |  |  |  | |
| 22 |  |  |  | |
| 23 |  |  |  | |
| 24 |  |  |  | |
| 25 |  |  |  | |
| 26 |  |  |  | |
| 27 |  |  |  | |
| 28 |  |  |  | |
| 29 |  |  |  | |
| 30 |  |  |  | |
| 31 |  |  |  | |
| 32 |  |  |  | |
| 33 |  |  |  | |
| 34 |  |  |  | |
| 35 |  |  |  | |
| 36 |  |  |  | |
| 37 |  |  |  | |
| 38 |  |  |  | |
| 39 |  |  |  | |
| 40 |  |  |  | |
| 41 |  |  |  | |
| 42 |  |  |  | |
| 43 |  |  |  | |
| 44 |  |  |  | |
| 45 |  |  |  | |
| 46 |  |  |  | |
| 47 |  |  |  | |
| 48 |  |  |  | |
| 49 |  |  |  | |
| 50 |  |  |  | |
| 51 |  |  |  | |
| 52 |  |  |  | |
| 53 |  |  |  | |
| 54 |  |  |  | |
| 55 |  |  |  | |
| 56 |  |  |  | |
| 57 |  |  |  | |
| 58 |  |  |  | |
| 59 |  |  |  | |
| 60 |  |  |  | |
| 61 |  |  |  | |
| 62 |  |  |  | |
| 63 |  |  |  | |
| 64 |  |  |  | |
| 65 |  |  |  | |
| 66 |  |  |  | |
| 67 |  |  |  | |
| 68 |  |  |  | |
| 69 |  |  |  | |
| 70 |  |  |  | |
| 71 |  |  |  | |
| 72 |  |  |  | |
| 73 |  |  |  | |
| 74 |  |  |  | |
| 75 |  |  |  | |
| 76 |  |  |  | |
| 77 |  |  |  | |
| 78 |  |  |  | |
| 79 |  |  |  | |
| 80 |  |  |  | |
| 81 |  |  |  | |
| 82 |  |  |  | |
| 83 |  |  |  | |
| 84 |  |  |  | |
| 85 |  |  |  | |
| 86 |  |  |  | |
| 87 |  |  |  | |
| 88 |  |  |  | |
| 90 |  |  |  | |

**PART 2: DEVELOP A FLOW CHART**

**Materials**

- In-depth process mapping worksheet
- Loose sheets of A4 paper
- Writing materials (pencils preferred)
- Markers (3-4 colours)
- (6 colours – green, pink, orange, blue, purple, yellow)
- Tape

**Instructions**

**Step 1:** Each group should put the activities identified in Part 1 in order by arranging the sticky notes on the flip chart paper in the proper sequence of when they should be conducted and drawing arrows between them.

- Start by writing the outcome (high MDA treatment coverage) in the center of the flipchart in red marker.
- Start with one category of activities and start placing the activities on the paper, keeping in mind the order in which they occur (remember we aim to depict the “flow” of activities).
- Be sure to leave space between the sticky notes (at least 3 finger widths). The group may need to tape several flip chart sheets together in order to fit all of the activities.
- Once all of the sticky notes have been placed on the flip chart, draw arrows between the sticky notes with a marker to show the desired sequence of activities.
- This should be repeated for every activity category, and arrows should be drawn between relationship categories, as relevant. A partially complete example is provided on the following page for reference.

**Step 2:** While creating the flow chart diagram, if the group identifies more activities that must take place in the delivery of MDA with high treatment coverage, please go back and add them to Table 1 and also create sticky notes of the relevant colour.

**Step 3:** When the process map is complete, the group should identify ten activities from each of the completed maps (one each for the intervention and control clusters) that are *essential* for achieving high coverage. Draw a star or circle on the sticky note of those ten activities.

**Step 4:** When the process map is finalized, the cluster leader should tape each sticky note down on the flip chart and take a picture of the map so that the map can be shared and further analysed.


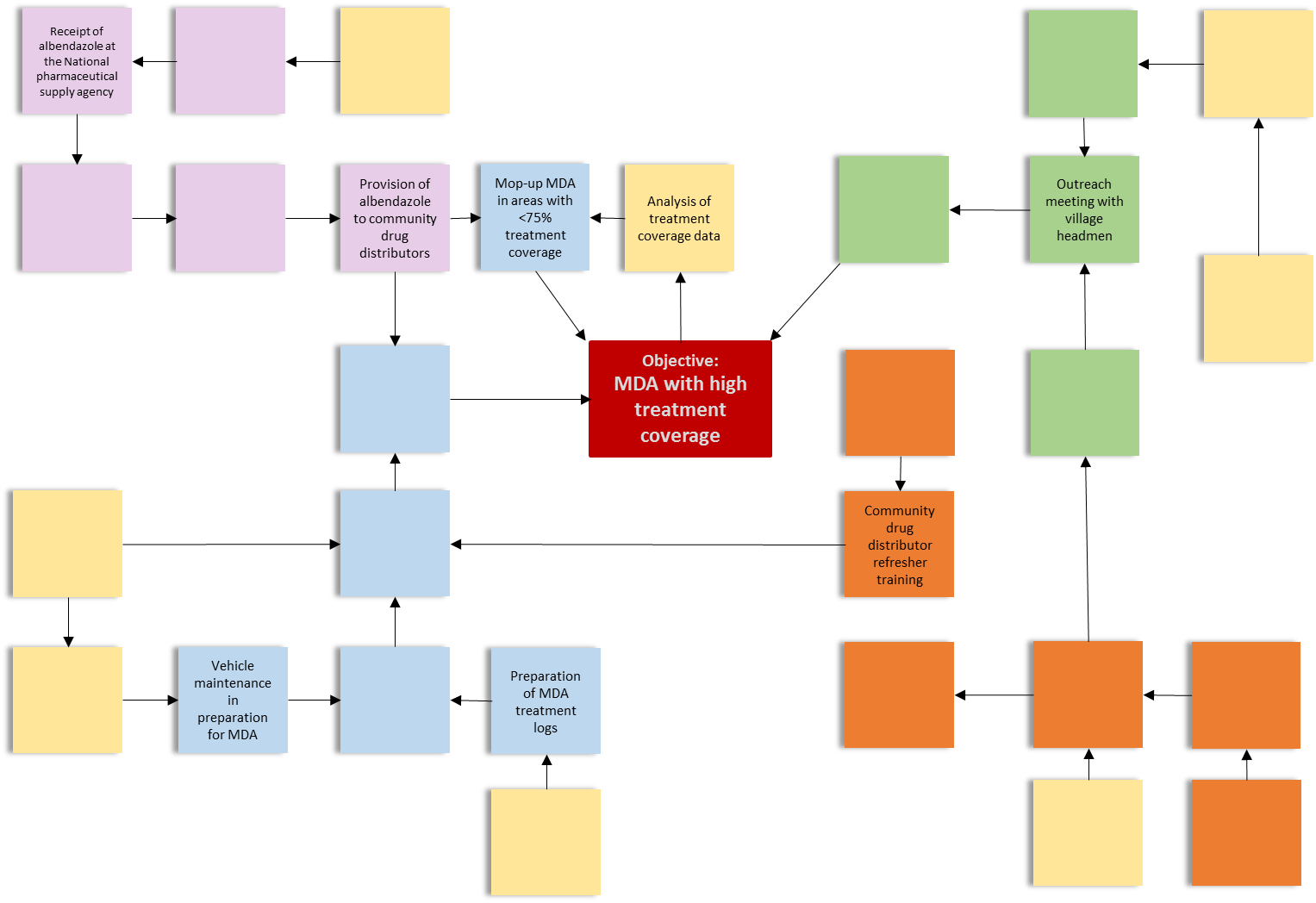
**Example of partially completed process map in a DeWorm3 intervention cluster**Note: There will be many more sticky notes (i.e. activities) in a completed process map

**PART 3: SETTING ACTIVITY TARGETS**

**Materials**

- Table 2 from in-depth process mapping worksheet
- Loose sheets of A4 paper
- Writing materials (pencils preferred)

**Instructions**

**Step 1:** After all of the activities and the flow of activities have been identified, please write the *ideal* targeted goal (i.e. metric) for each completed activity.

- Be specific with the targets – quantify each target goal as shown in the example below.
- Keep the goals very brief (1-2 sentences) as the purpose is for individuals in other settings to understand generally the cluster’s ideal metrics for each activity.
- If more space is needed, please use an additional piece of paper or make photocopies of Table 2.

**Step 2:** Write the *ideal* targeted timeline for when each activity should be completed.

- Be specific with the timeline as shown in the example below.
- Keep the timelines very brief (1-2 sentences).

**Step 3:** One of the DeWorm3 Project’s goals is to understand what aspects of the previous lymphatic filariasis (LF) program are utilized for the delivery of community-wide delivery of STH MDA. This could include existing supply chains, personnel, communication channels, etc. In the last column of Table 3, please record if an activity leverages part of the previous LF platform. If it does, please briefly write (1-2 sentences) describing how the previous LF platform is being built upon to support community-wide delivery of STH MDA.

**Step 4:** The completed Table 1, the completed Table 2, the picture of the process map, and the original process map with sticky notes taped onto flip chart paper should be returned to the local DeWorm3 office, where the data manager will enter them into the SurveyCTO database.

| **Table 2: Activity Targets and Goals** | | |  | | |  |
| --- | --- | --- | --- | --- | --- | --- |
|  |  | **Step 1** | | **Step 2** | **Step 3** | |
| **#** | **Activity** | **Target Metric** | | **Target timeline** | **Is the LF platform being leveraged for this activity?** | |
|  |  |  |  |  | *Yes/No* | *If yes, description:* |
| 1 | *Example: Contact village leaders to notify about upcoming MDA campaign* | *100% of village heads notified of upcoming MDA during in-person meeting* | | *One month in advance of MDA start date.* | 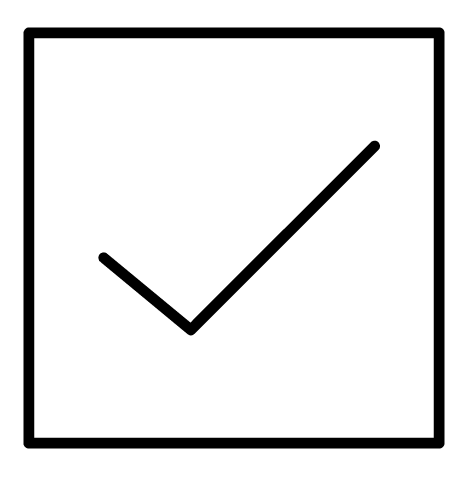 Yes  No | *Will use the LF programme’s community contact database from 2012* |
| 2 |  |  | |  | Yes  No |  |
| 3 |  |  | |  | Yes  No |  |
| 4 |  |  | |  | Yes  No |  |
| 5 |  |  | |  | Yes  No |  |
| 6 |  |  | |  | Yes  No |  |
| 7 |  |  | |  | Yes  No |  |
| 8 |  |  | |  | Yes  No |  |
| 9 |  |  | |  | Yes  No |  |
| 10 |  |  | |  | Yes  No |  |
| 11 |  |  | |  | Yes  No |  |
| 12 |  |  | |  | Yes  No |  |
| 13 |  |  | |  | Yes  No |  |
| 14 |  |  | |  | Yes  No |  |
| 15 |  |  | |  | Yes  No |  |
| 16 |  |  | |  | Yes  No |  |
| 17 |  |  | |  | Yes  No |  |
| 18 |  |  | |  | Yes  No |  |
| 19 |  |  | |  | Yes  No |  |
| 20 |  |  | |  | Yes  No |  |
| 21 |  |  | |  | Yes  No |  |
| 22 |  |  | |  | Yes  No |  |
| 23 |  |  | |  | Yes  No |  |
| 24 |  |  | |  | Yes  No |  |
| 25 |  |  | |  | Yes  No |  |
| 26 |  |  | |  | Yes  No |  |
| 27 |  |  | |  | Yes  No |  |
| 28 |  |  | |  | Yes  No |  |
| 29 |  |  | |  | Yes  No |  |
| 30 |  |  | |  | Yes  No |  |
| 31 |  |  | |  | Yes  No |  |
| 32 |  |  | |  | Yes  No |  |
| 33 |  |  | |  | Yes  No |  |
| 34 |  |  | |  | Yes  No |  |
| 35 |  |  | |  | Yes  No |  |
| 36 |  |  | |  | Yes  No |  |
| 37 |  |  | |  | Yes  No |  |
| 38 |  |  | |  | Yes  No |  |
| 39 |  |  | |  | Yes  No |  |
| 40 |  |  | |  | Yes  No |  |
| 41 |  |  | |  | Yes  No |  |
| 42 |  |  | |  | Yes  No |  |
| 43 |  |  | |  | Yes  No |  |
| 44 |  |  | |  | Yes  No |  |
| 45 |  |  | |  | Yes  No |  |
| 46 |  |  | |  | Yes  No |  |
| 47 |  |  | |  | Yes  No |  |
| 48 |  |  | |  | Yes  No |  |
| 49 |  |  | |  | Yes  No |  |
| 50 |  |  | |  | Yes  No |  |
| 51 |  |  | |  | Yes  No |  |
| 52 |  |  | |  | Yes  No |  |
| 53 |  |  | |  | Yes  No |  |
| 54 |  |  | |  | Yes  No |  |
| 55 |  |  | |  | Yes  No |  |
| 56 |  |  | |  | Yes  No |  |
| 57 |  |  | |  | Yes  No |  |
| 58 |  |  | |  | Yes  No |  |
| 59 |  |  | |  | Yes  No |  |
| 60 |  |  | |  | Yes  No |  |
| 61 |  |  | |  | Yes  No |  |
| 62 |  |  | |  | Yes  No |  |
| 63 |  |  | |  | Yes  No |  |
| 64 |  |  | |  | Yes  No |  |
| 65 |  |  | |  | Yes  No |  |
| 66 |  |  | |  | Yes  No |  |
| 67 |  |  | |  | Yes  No |  |
| 68 |  |  | |  | Yes  No |  |
| 69 |  |  | |  | Yes  No |  |
| 70 |  |  | |  | Yes  No |  |
| 71 |  |  | |  | Yes  No |  |
| 72 |  |  | |  | Yes  No |  |
| 73 |  |  | |  | Yes  No |  |
| 74 |  |  | |  | Yes  No |  |
| 75 |  |  | |  | Yes  No |  |
| 76 |  |  | |  | Yes  No |  |
| 77 |  |  | |  | Yes  No |  |
| 78 |  |  | |  | Yes  No |  |
| 79 |  |  | |  | Yes  No |  |
| 80 |  |  | |  | Yes  No |  |
| 81 |  |  | |  | Yes  No |  |
| 82 |  |  | |  | Yes  No |  |
| 83 |  |  | |  | Yes  No |  |
| 84 |  |  | |  | Yes  No |  |
| 85 |  |  | |  | Yes  No |  |
| 86 |  |  | |  | Yes  No |  |
| 87 |  |  | |  | Yes  No |  |
| 88 |  |  | |  | Yes  No |  |
| 89 |  |  | |  | Yes  No |  |
| 90 |  |  | |  | Yes  No |  |

**PART 4: IN-DEPTH PROCESS MAPPING UPDATE (completed 6-12 months after Parts 1-3)**

**Materials**

- Post-MDA Activity Progress Table
- Writing materials (pen or pencil)

**Instructions**

- Identify the individual from this working group who will be responsible for tracking the progress of each of these activities as they occur. This person is the “cluster process mapping leader”. This person will be responsible for completing Table 3 annually.

Cluster process mapping lead name: _______________________________

Job title & affiliation: __________________________________________________________

Primary phone number: __________________________ Email: __________________________

- Observed activities should be recorded in Table 3 at the end of year one, year two, and year three.
  - In intervention clusters, the update will occur following the second round of MDA, the fourth round of MDA, and the sixth round of biannual MDA.
  - In control clusters, the update will occur after the first, second, and third rounds of annual MDA
- A typed version of Table 3 will be provided to the process mapping lead to update each year. Columns A, B, and C will already be completed in the typed table, thus they will not need to re-write the activities, metrics, and timelines identified during the baseline working group meeting.
- In order to avoid inaccuracies, activity progress should be recorded in real-time. DO NOT wait until the end of the year to update the form.
- It is very important to remember that the purpose of this exercise is not to identify problems in delivery, but rather to understand how MDA delivery occurs in reality, and how this does or does not influence MDA treatment coverage.
- The completed table will be returned to the DeWorm3 central office within one month of MDA.

| **Table 3: Post-MDA Observed Activity Progress  (NOTE: This is an example. Tables will be provided to process mapping leads with columns A-C pre-populated)** | | | | | | |
| --- | --- | --- | --- | --- | --- | --- |
|  | **A** | **B** | **C** | **D** | **E** | **F** |
| **#** | **Activity** | **Target Goal** | **Target time** | **Observed progress** | **Observed timeline** | **Reason for deviation between target/observed** |
| 1 | *Example: Meeting with village headmen* | *100% of village heads notified of upcoming MDA during in-person meeting* | *One month in advance of MDA start date.* | *65% of village headmen were notified at a meeting at the Chief’s house* | *5 weeks in advance of MDA.* | *Many headmen attended an agricultural event in Shaya Village and could not participate in the DeWorm3 meeting.* |
| 2 |  |  |  |  |  |  |
| 3 |  |  |  |  |  |  |
| 4 |  |  |  |  |  |  |
| 5 |  |  |  |  |  |  |
| 6 |  |  |  |  |  |  |
| 7 |  |  |  |  |  |  |
| 8 |  |  |  |  |  |  |
| 9 |  |  |  |  |  |  |
