# Supplementary material for: Identifying opportunities to optimize mass drug administration for soil-transmitted helminths: A visualization and descriptive analysis using process mapping
Source: PLoS Negl Trop Dis. 2024 Jan 4;18(1):e0011772. doi: 10.1371/journal.pntd.0011772 (PMC10793904; doi:10.1371/journal.pntd.0011772)
Supplement: S1 Table — Proportion of activities in each activity category, by cluster. (DOCX) [file pntd.0011772.s002.docx]

|  | Cluster Characteristics | | | Proportion of Activities, by category (%) | | | | | | |
| --- | --- | --- | --- | --- | --- | --- | --- | --- | --- | --- |
|  | Intervention^1^ | Historical Coverage Level^2^ | Number of activities | Planning | Drug Supply Chain | Training | Community Sensitization | MDA Delivery | M&E | Other |
| Cluster A | cMDA | low | 32 | 2 (6.3%) | 12 (37.5%) | 8 (25.0%) | 8 (25.0%) | 2 (6.3%) | 0 (0.0%) | 0 (0.0%) |
| Cluster B | cMDA | high | 31 | 6 (19.4%) | 6 (19.4%) | 4 (12.9%) | 6 (19.4%) | 4 (12.9%) | 2 (6.5%) | 3 (9.7%) |
| Cluster C | cMDA | high | 36 | 7 (19.4%) | 7 (19.4%) | 2 (5.6%) | 9 (25.0%) | 5 (13.9%) | 6 (16.7%) | 0 (0.0%) |
| Cluster D | cMDA | high | 27 | 5 (18.5%) | 5 (18.5%) | 2 (7.4%) | 6 (22.2%) | 3 (11.1%) | 6 (22.2%) | 0 (0.0%) |
| Cluster E | school-based | high | 28 | 7 (25.0%) | 2 (7.1%) | 2 (7.1%) | 5 (17.9%) | 6 (21.4%) | 6 (21.4%) | 0 (0.0%) |
| Cluster F | school-based | low | 31 | 7 (22.6%) | 7 (22.6%) | 2 (6.5%) | 5 (16.1%) | 5 (16.1%) | 5 (16.1%) | 0 (0.0%) |
| Cluster G | cMDA | low | 34 | 8 (23.5%) | 6 (17.6%) | 3 (8.8%) | 7 (20.6%) | 3 (8.8%) | 7 (20.6%) | 0 (0.0%) |
| Cluster H | cMDA | low | 27 | 2 (7.4%) | 4 (14.8%) | 4 (14.8%) | 7 (25.9%) | 4 (14.8%) | 5 (18.5%) | 1 (3.7%) |
| Cluster I | cMDA | high | 30 | 2 (6.7%) | 8 (26.7%) | 7 (23.3%) | 7 (23.3%) | 5 (16.7%) | 1 (3.3%) | 0 (0.0%) |
| Cluster J | school-based | low | 26 | 5 (19.2%) | 4 (15.4%) | 6 (23.1%) | 4 (15.4%) | 4 (15.4%) | 3 (11.5%) | 0 (0.0%) |
| Cluster K | school-based | high | 49 | 22 (44.9%) | 7 (14.3%) | 5 (10.2%) | 6 (12.2%) | 4 (8.2%) | 4 (8.2%) | 1 (2.0%) |
| Cluster L | cMDA | low | 27 | 4 (14.8%) | 5 (18.5%) | 5 (18.5%) | 5 (18.5%) | 7 (25.9%) | 1 (3.7%) | 0 (0.0%) |
| Cluster M | cMDA | low | 72 | 19 (26.4%) | 18 (25.0%) | 7 (9.7%) | 18 (25.0%) | 7 (9.7%) | 3 (4.2%) | 0 (0.0%) |
| Cluster N | cMDA | high | 82 | 32 (39.0%) | 21 (25.6%) | 4 (4.9%) | 15 (18.3%) | 10 (12.2%) | 0 (0.0%) | 0 (0.0%) |
| Cluster O | school-based | high | 19 | 4 (21.1%) | 5 (26.3%) | 2 (10.5%) | 3 (15.8%) | 4 (21.1%) | 1 (5.3%) | 0 (0.0%) |
| Cluster P | cMDA | high | 91 | 41 (45.1%) | 21 (23.1%) | 5 (5.5%) | 12 (13.2%) | 12 (13.2%) | 0 (0.0%) | 0 (0.0%) |
| Cluster Q | cMDA | low | 81 | 33 (40.7%) | 21 (25.9%) | 5 (6.2%) | 13 (16.0%) | 9 (11.1%) | 0 (0.0%) | 0 (0.0%) |
| Cluster R | school-based | low | 53 | 18 (34.0%) | 25 (47.2%) | 5 (9.4%) | 1 (1.9%) | 4 (7.5%) | 0 (0.0%) | 0 (0.0%) |
| ^1^School-based MDA includes data from 6 clusters and cMDA includes data from 12 clusters  ^2^ Historically high coverage clusters had over 80% coverage historically low coverage had below 60% coverage | | | | | | | | | | |
