# Supplementary material for: Identifying opportunities to optimize mass drug administration for soil-transmitted helminths: A visualization and descriptive analysis using process mapping
Source: PLoS Negl Trop Dis. 2024 Jan 4;18(1):e0011772. doi: 10.1371/journal.pntd.0011772 (PMC10793904; doi:10.1371/journal.pntd.0011772)
Supplement: S2 Table — Total adaptations and percent change in number of activities compared to previous round for each cluster. (DOCX) [file pntd.0011772.s003.docx]

|  | Total Adaptations | | Percent change in number of activities from previous round | | |
| --- | --- | --- | --- | --- | --- |
|  | Average per round | Total over three years | Year 1 | Year 2 | Year 3 |
| Cluster A | 6.0 | 18 | 25.0% | 7.5% | 7.0% |
| Cluster B | 5.0 | 15 | 9.7% | 17.6% | 10.0% |
| Cluster C | 4.0 | 12 | -11.1% | 25.0% | 0.0% |
| Cluster D | 5.7 | 17 | -22.2% | 28.6% | -3.7% |
| Cluster E | 4.7 | 14 | -17.9% | 0.0% | -13.0% |
| Cluster F | 3.3 | 10 | -16.1% | -3.8% | 0.0% |
| Cluster G | 4.7 | 14 | -11.8% | -6.7% | 0.0% |
| Cluster H | 6.0 | 18 | -29.6% | 42.1% | 7.4% |
| Cluster I | 5.0 | 15 | 23.3% | 0.0% | 5.4% |
| Cluster J | 3.0 | 9 | 26.9% | 3.0% | -2.9% |
| Cluster K | 2.0 | 6 | -8.2% | -4.4% | 0.0% |
| Cluster L | 5.3 | 16 | 33.3% | 2.8% | 0.0% |
| Cluster M | 2.3 | 7 | -1.4% | 0.0% | 0.0% |
| Cluster N | 6.0 | 18 | -12.2% | -2.8% | 0.0% |
| Cluster O | 1.0 | 3 | 10.5% | 0.0% | 4.8% |
| Cluster P | 6.7 | 20 | -11.0% | -4.9% | 0.0% |
| Cluster Q | 5.3 | 16 | -12.3% | 0.0% | 0.0% |
| Cluster R | 2.3 | 7 | -7.5% | -6.1% | 0.0% |
