# Supplementary material for: Identifying opportunities to optimize mass drug administration for soil-transmitted helminths: A visualization and descriptive analysis using process mapping
Source: PLoS Negl Trop Dis. 2024 Jan 4;18(1):e0011772. doi: 10.1371/journal.pntd.0011772 (PMC10793904; doi:10.1371/journal.pntd.0011772)
Supplement: S3 Table — Average deviations per round and proportion of activities with goal and time deviations by a number of cluster and activity characteristics. (DOCX) [file pntd.0011772.s004.docx]

|  | Total Deviations | | | | Proportion of Activities with Deviations | | | | | |
| --- | --- | --- | --- | --- | --- | --- | --- | --- | --- | --- |
|  | Average per round | | Total over three rounds | | Year 1 | | Year 2 | | Year 3 | |
|  | Goal | Time | Goal | Time | Goal | Time | Goal | Time | Goal | Time |
| **Country** | | | | | | | | | | |
| Benin | 2.7 | 10.3 | 48 | 185 | 9.3% | 32.7% | 5.9% | 32.7% | 9.9% | 31.1% |
| India | 7.4 | 28.7 | 133 | 517 | 6.2% | 30.6% | 7.4% | 57.4% | 21.7% | 49.8% |
| Malawi | 4.6 | 11.1 | 73 | 178 | 16.3% | 42.4% | 24.8% | 52.6% | 14.5% | 40.4% |
| **Intervention**^1^ | | | | | | | | | | |
| School-based | 6.4 | 11.4 | 102 | 182 | 10.1% | 24.8% | 14.5% | 50.0% | 33.0% | 30.3% |
| Community-wide | 4.3 | 8.1 | 152 | 698 | 8.0% | 34.8% | 8.9% | 49.2% | 11.5% | 46.6% |
| **Historical coverage**^2^ | | | | | | | | | | |
| Low coverage | 5.4 | 17.5 | 139 | 455 | 8.2% | 35.7% | 10.9% | 49.9% | 19.4% | 42.8% |
| High coverage | 4.4 | 16.3 | 115 | 425 | 8.6% | 29.9% | 9.8% | 49.0% | 14.2% | 42.2% |
| **Category** | | | | | | | | | | |
| Planning | 1.4 | 5.4 | 73 | 279 | 7.2% | 27.3% | 13.0% | 64.9% | 18.9% | 57.3% |
| Drug Supply Chain | 0.8 | 3.8 | 42 | 200 | 9.2% | 36.2% | 3.6% | 45.8% | 13.3% | 41.6% |
| Training | 0.6 | 2.6 | 30 | 137 | 4.2% | 60.6% | 10.8% | 57.8% | 21.7% | 55.4% |
| Community Sensitization | 0.9 | 3.9 | 45 | 203 | 10.3% | 42.7% | 7.9% | 57.1% | 18.3% | 64.3% |
| MDA Delivery | 0.9 | 0.7 | 46 | 36 | 7.0% | 8.1% | 16.8% | 15.0% | 18.6% | 10.6% |
| M&E | 0.3 | 0.5 | 15 | 24 | 21.1% | 15.8% | 14.3% | 50.0% | 25.0% | 25.0% |
| Other | 0.1 | 0.0 | 3 | 1 | 25.0% | 0.0% | 33.3% | 33.3% | 33.3% | 0.0% |
| **Time** | | | | | | | | | | |
| Up to 2 months before MDA | 0.6 | 1.6 | 29 | 82 | 6.4% | 31.7% | 10.0% | 51.7% | 31.7% | 51.7% |
| 2 months – 2 weeks before MDA | 1.3 | 8.4 | 70 | 439 | 8.2% | 40.6% | 9.8% | 63.9% | 9.4% | 67.5% |
| 2 weeks – beginning of MDA | 1.3 | 5.4 | 69 | 279 | 7.9% | 41.2% | 5.0% | 55.5% | 22.5% | 47.5% |
| During MDA | 0.9 | 0.3 | 46 | 14 | 7.3% | 1.0% | 16.5% | 8.3% | 15.7% | 2.5% |
| After MDA | 0.8 | 1.3 | 40 | 66 | 15.7% | 25.5% | 17.6% | 48.5% | 29.4% | 29.4% |
| **Deviation Category** | | | | | | | | | | |
| Purposeful Change: efficiency | 0.9 | 4.9 | 48 | 257 | 2.0% | 4.2% | 4.1% | 18.3% | 0.8% | 13.4% |
| Purposeful Change: effectiveness | 0.2 | 2.8 | 12 | 145 | 0.5% | 3.7% | 1.0% | 10.7% | 0.3% | 6.1% |
| Competing Priorities or Dependency Delays | 0.4 | 3.7 | 23 | 190 | 1.2% | 12.9% | 1.0% | 11.6% | 1.1% | 3.3% |
| Community Influences | 0.6 | 0.8 | 33 | 42 | 0.9% | 4.0% | 3.0% | 2.1% | 0.8% | 0.1% |
| Resource Constraints | 0.4 | 0.4 | 20 | 19 | 1.9% | 0.5% | 0.4% | 1.6% | 0.7% | 0.7% |
| MoH or National Program Linkages | 0.3 | 1.8 | 15 | 93 | 0.6% | 3.6% | 0.1% | 2.8% | 1.3% | 6.6% |
| Covid-19 | 4.0 | 8.2 | 72 | 148 | - | - | - | - | 9.5% | 19.6% |
| Other | 0.7 | 1.3 | 37 | 66 | 1.7% | 2.5% | 0.7% | 2.1% | 3.2% | 4.6% |
| ^1^School-based MDA includes data from 6 clusters and cMDA includes data from 12 clusters  ^2^Historically high coverage clusters had over 80% coverage historically low coverage had below 60% coverage | | | | | | | | | | |
