# Supplementary material for: Identifying opportunities to optimize mass drug administration for soil-transmitted helminths: A visualization and descriptive analysis using process mapping
Source: PLoS Negl Trop Dis. 2024 Jan 4;18(1):e0011772. doi: 10.1371/journal.pntd.0011772 (PMC10793904; doi:10.1371/journal.pntd.0011772)
Supplement: S4 Table — Average goal and time deviations and proportion of activities with goal and time deviations at each update, for each cluster. (DOCX) [file pntd.0011772.s005.docx]

##

|  | Total Deviations | | | | Proportion of Activities with Deviations | | | | | |
| --- | --- | --- | --- | --- | --- | --- | --- | --- | --- | --- |
|  | Average per round | | Total over three rounds | | Year 1 | | Year 2 | | Year 3 | |
|  | Goal | Time | Goal | Time | Goal | Time | Goal | Time | Goal | Time |
| Cluster A | 4.0 | 19.3 | 12 | 58 | 9.7% | 58.1% | 10.0% | 27.5% | 11.6% | 67.4% |
| Cluster B | 3.7 | 6.7 | 11 | 20 | 6.7% | 20.0% | 8.8% | 23.5% | 15.0% | 15.0% |
| Cluster C | 5.7 | 14.7 | 17 | 44 | 15.6% | 46.9% | 25.0% | 40.6% | 10.0% | 40.0% |
| Cluster D | 4.0 | 10.3 | 12 | 31 | 9.5% | 42.9% | 31.6% | 57.9% | 15.4% | 42.3% |
| Cluster E^1^ | 2.5 | 9.0 | 5 | 18 | - | - | 15.0% | 45.0% | 10.0% | 45.0% |
| Cluster F^1^ | 6.5 | 12.5 | 13 | 25 | - | - | 47.8% | 56.5% | 8.0% | 48.0% |
| Cluster G | 6.3 | 9.7 | 19 | 29 | 26.7% | 33.3% | 12.5% | 54.2% | 28.6% | 21.4% |
| Cluster H | 2.3 | 10.3 | 7 | 31 | 0.0% | 26.3% | 15.8% | 68.4% | 14.8% | 48.1% |
| Cluster I | 2.0 | 11.3 | 6 | 34 | 10.3% | 24.1% | 2.7% | 43.2% | 5.4% | 29.7% |
| Cluster J | 4.0 | 10.0 | 12 | 30 | 19.2% | 26.9% | 0.0% | 39.4% | 21.2% | 30.3% |
| Cluster K | 8.7 | 11.3 | 26 | 34 | 6.7% | 2.2% | 7.0% | 41.9% | 46.5% | 34.9% |
| Cluster L | 1.7 | 7.0 | 5 | 21 | 3.7% | 25.9% | 8.3% | 25.0% | 2.7% | 13.5% |
| Cluster M | 5.3 | 33.0 | 16 | 99 | 5.6% | 38.0% | 4.4% | 51.5% | 12.7% | 52.1% |
| Cluster N | 5.7 | 40.0 | 17 | 120 | 9.7% | 47.2% | 3.0% | 65.7% | 11.4% | 60.0% |
| Cluster O | 0.7 | 7.3 | 2 | 22 | 5.3% | 42.1% | 4.8% | 42.9% | 0.0% | 23.8% |
| Cluster P | 6.3 | 34.0 | 19 | 102 | 6.2% | 21.0% | 9.5% | 56.8% | 9.1% | 55.8% |
| Cluster Q | 3.7 | 36.3 | 11 | 109 | 1.4% | 33.8% | 4.4% | 58.8% | 9.9% | 63.4% |
| Cluster R | 14.7 | 17.7 | 44 | 53 | 8.2% | 32.7% | 19.6% | 67.4% | 67.4% | 13.0% |

^1^Cluster E and F did not submit data at the Year 1 update
