# Supplementary material for: Identifying opportunities to optimize mass drug administration for soil-transmitted helminths: A visualization and descriptive analysis using process mapping
Source: PLoS Negl Trop Dis. 2024 Jan 4;18(1):e0011772. doi: 10.1371/journal.pntd.0011772 (PMC10793904; doi:10.1371/journal.pntd.0011772)
Supplement: Attachment — Submitted filename: Review.docx [file pntd.0011772.s007.docx]

**Manuscript Title:**

Identifying opportunities to optimize mass drug administration for soil-transmitted

helminths: a visualization and descriptive analysis using process mapping

**Reviewer’s decision**

The manuscript is of high quality considering the context, relevance, amount of work-done, analysis made, and style of writing/presentation. However, I think it would be more beneficial to have the therapeutic coverage (the %), and category for each cluster tabulated against the table on adaptation. This answers programmatic questions such as; were the adaptations improving coverage of medicines across the years. This line can also be captured in the result and discussion section (if there is a trend). This should also be performed for fidelity (i.e., deviations).

**Reviewer’s Comment**

**TOPIC:**

The title of this manuscript is appropriate and concise,

**INTRODUCTION**

Well detailed, sufficiently referenced, and rationale clearly spelt out

***Materials and Methods***

Line 124: authors should recast this line: and study design and implementation science research

**RESULTS**

Table 1; Authors should also update table 1 with all the chi-square values and p-values, just as they have done within the text. Also, this result is missing on the table “and historically high and

low coverage clusters (χ2 = 9.9, df = 6, p = 0.13)…..

Figure 1: This figure currently masks a lot of information (% of deviations). I suggest authors converts this to a table and include coverage estimates as stated in my general comments above.

**Discussion:**

Line 438: please replace “to try to” with “to”
