# Supplementary material for: Identifying opportunities to optimize mass drug administration for soil-transmitted helminths: A visualization and descriptive analysis using process mapping
Source: PLoS Negl Trop Dis. 2024 Jan 4;18(1):e0011772. doi: 10.1371/journal.pntd.0011772 (PMC10793904; doi:10.1371/journal.pntd.0011772)
Supplement: Attachment — Submitted filename: DeWorm 3 PM - Reviewer response letter - 2023_06_27.docx [file pntd.0011772.s008.docx]

May 31, 2023

Uwem Friday Ekpo and Francesca Tamarozzi

Editors

PLOS Neglected Tropical Diseases

Re: PLOS Neglected Tropical Diseases submission (PNTD-D-23-00169)

Dear Dr. Ekpo and Dr. Tamarozzi,

We would like to sincerely thank you for reviewing and providing comments regarding our article entitled: "Identifying opportunities to optimize mass drug administration for soil-transmitted helminths: a visualization and descriptive analysis using process mapping". We have reviewed the editorial and reviewer comments and modified the manuscript in response to the reviewer feedback. Below, you will find the comments and our point-by-point responses (in bold).

*METHODS*

Reviewer #2:

- Line 124: authors should recast this line: and study design and implementation science research. **This line has been simplified and now reads “The DeWorm3 trial rationale and study design are described in detail elsewhere (28,29).” The references here are to the DeWorm3 trial protocol and the DeWorm 3 implementation research protocol.**

----------

*RESULTS*

Reviewer #2:

- Table 1; Authors should also update table 1 with all the chi-square values and p-values, just as they have done within the text. Also, this result is missing on the table “and historically high and low coverage clusters (χ2 = 9.9, df = 6, p = 0.13).

**Chi-square tests were performed to identify differences in the distribution of activity categories between delivery platforms and historical coverage levels, and whether cMDA had a larger proportion of community sensitization activities than school-based MDA. Since chi-square tests were not performed for all variables included in Table 1 (driven by a prior hypotheses and variable structures) we have elected to display results in the text rather than in the table. We have added the comparison between historically high and low coverage clusters to Table 1. Deviation data comparing historically high and low coverage clusters is available in S3 Table.**

- Figure 1: This figure currently masks a lot of information (% of deviations). I suggest authors converts this to a table and include coverage estimates as stated in my general comments above.

**Thank you for this suggestion. We are cognizant that this manuscript presents a large amount of data, which can be challenging to digest. We created Figure 1 in an attempt to highlight most notable comparisons and share these comparisons in a more visual format. However, we agree with the reviewer that Figure 1 does not present all information; we have included a table which reports all deviation data as a supplementary file (S3 Table). We have highlighted S3 more prominently in the text.**

----------

*DISCUSSION*

Reviewer #2:

- Line 438: please replace “to try to” with “to”.

**This change has been made.**

----------

*GENERAL COMMENTS*

Reviewer #1:

- I understand focus on high and low coverage. However, many clusters will fall into the moderate category (60-80%). Do you think they will differ at all? Do you have an estimate for what proportion of clusters this represents?

**The reviewer is correct that many clusters had historically moderate coverage (between 60 and 80%). We sampled clusters with historically higher and lower coverage to ensure there was intentionally varied sampling in the event that there were indeed meaningful differences. We also aimed to learn about any differences in processes between settings where MDA has had higher and lower coverage. Interestingly, our analysis demonstrated minimal difference in baseline plans, adaptations, and fidelity to plans between historical coverage levels. In the trial environment, coverage levels were generally high, even in clusters with historically low coverage.**

- I agree with the emphasis that deviations are not necessarily negative and changes can be purposeful. In general, they can be a sign of good adaptive management. Was / is it possible to collect information as to whether changes were intentional or forced?

**It is certainly possible to collect data around whether deviations were planned or unplanned. In fact, our study presents reasons for deviations, including if the deviations were purposeful or non-purposeful.**

- Likewise, would it be possible to record whether changes were viewed as having a positive or negative impact?

**It would be possible to collect this information, with careful attention to how “impact” is defined. We did not include this in the current research. We found that best practice was to collect PM data immediately following MDA, and perhaps before the full impacts of adaptations could be observed. With additional data collection activities, it would be possible to make this assessment.**

- I think this is a very important concept: “Building organizational cultures that normalize mid-course adjustments and iterative planning(42) may be particularly well suited to settings transitioning from a school-based to community-wide campaign approaches.” To this end, would it be possible to collect information on whether individuals were comfortable suggesting / implementing changes if they felt it would improve the program, rather than slavishly sticking to the plan?

**We appreciate this very interesting idea from the reviewer. This could be collected within the PM updates, however this and the aforementioned additional data points may be better suited to in depth interviews. We did encounter some challenges with PM data collection as it can be quite labor intensive to review the entire MDA process in this highly granular way, activity by activity. We recommend that “PM may need to be simplified to match resource constraints of a particular national or sub-national campaign if used routinely in the future.”**

- You report that the number of planning activities remains high even in Year 3. Could this also reflect high turnover of personnel?

**This is possible, however many of the planning activities that remained in the MDA process through the trial were repeated at each round. For instance, setting dates for MDA and informing local leaders are planning activities that happen at each and every round.**

- Why do you think there were so many more activities in India than other countries? Is this related to how they are named / described, or something deeper?

**We believe there are multiple reasons:**

- **How they are named/described – in India, activities were consistently identified at a more granular level than in Benin and Malawi. This is indicated in lines 439-442 of the manuscript.**
- **The government structure in India has more levels and there are more unique roles cited in PM activities than in Benin or Malawi. Many activities in India describe information or supply chain connections between the multi-layered actors in the public health system that delivers MDA.**
- Figure 2 – Process Map. Many of these activities will likely be happening concurrently, rather than in a neat step-wise manner. Is PM able to capture that?

**PM is able to capture some simple concurrences, however as the examples get more complex, the visualizations becomes more challenging to read or understand. We believe some of these timing details are well captured in the timing categories presenting, including up to 2 months before MDA, 2 months to 2 weeks before MDA, 2 weeks before the start of MDA, during MDA, or after MDA. A Gantt chart may be a better way of understanding activity concurrences and overlaps, however this would be a complementary tool to a process map depicting flow.**

- You mention the importance of microplanning. Of course, the WHO have just released an NTD Microplanning Guide and training modules – are there explicit links to this in that guide?

**Thank you for this comment. Yes, PM may be a very useful tool to support implementation of these recently released guidelines, especially around Step 4. This has been added to the manuscript in lines 103-105: “This can inform microplanning such as that described in the WHO’s recently released microplanning guide which indicates the importance of “plan[ning] the activities required to reach and treat target populations” (20.”**

- In many countries we are approaching / aiming for the end of standalone STH programs, and rather having them mainstreamed into MOH / government health systems. Can the same process be used in those situations? It’s referenced in lines 385-389 with respect to supply chain specifically, but also thinking more broadly than that.

**Absolutely – in fact PM may be particularly useful for identifying points of connection between STH and other NTD programs, or other community-based campaigns. This is reflected in lines 387-391: “In addition, the highly detailed view of the implementation cascade produced by PM may be used to identify areas of possible integration with other community-based campaigns. For example, understanding the steps in the drug supply chain for STH deworming may allow planners to realistically select other campaigns or commodities that could share supply chain timelines and resources.”**

Reviewer #2:

- The manuscript is of high quality considering the context, relevance, amount of work-done, analysis made, and style of writing/presentation. However, I think it would be more beneficial to have the therapeutic coverage (the % and category) tabulated against the table on adaptation. This answers programmatic questions such as; were the adaptations improving coverage of medicines across the years. This line can also be captured in the result and discussion section (if there is a trend). This should also be performed for fidelity (i.e., deviations).

**Thank you for this comment. In the trial environment, MDA coverage levels were uniformly high across all clusters and rounds. Because of this, we have elected to not include analysis of coverage levels for adaptation and fidelity. We are preparing an additional manuscript that will more thoroughly explore the potential relationship between fidelity and coverage, along with other potential drivers of coverage. We have added a line to the Methods section (200-202) to this effect: “****MDA treatment coverage was uniformly high across all clusters and rounds, and potential associations between coverage and adaptations or deviations will be reported in future manuscripts.”**

**We understand however that the reader will naturally want to understand any potential relationships between process and coverage, however these data have not yet been published, so we are unable to present them in this manuscript Work is ongoing on two future manuscripts (1) presents DeWorm3 coverage across a variety of coverage definitions for all trial clusters, and (2) analyzes the association between process adaptation/fidelity and coverage.**
